# SARS-CoV-2 Vaccination: What Can We Expect Now?

**DOI:** 10.3390/vaccines10071093

**Published:** 2022-07-08

**Authors:** François Meurens, Fanny Renois, Alexis Bouin, Jianzhong Zhu

**Affiliations:** 1 INRAE, Oniris, BIOEPAR, 44300 Nantes, France; fanny.renois@inrae.fr; 2 Department of Veterinary Microbiology and Immunology, Western College of Veterinary Medicine, University of Saskatchewan, Saskatoon, SK S7N 5E2, Canada; 3 Department of Microbiology & Molecular Genetics, School of Medicine, University of California, Irvine, CA 92697, USA; abouin@uci.edu; 4 College Veterinary Medicine, Yangzhou University, Yangzhou 225009, China; jzzhu@yzu.edu.cn; 5 Joint International Research Laboratory of Agriculture and Agri-Product Safety, Yangzhou University, Yangzhou 225009, China

At the beginning of summer 2022, my colleagues and I wanted to share some thoughts about a vaccination success story, i.e., the first messenger RNA (mRNA) vaccines against coronavirus disease 2019’s (COVID-19) [1,2]. Indeed, after a one-year vaccination campaign taking place in almost every country, the time has come to think about what we could see next in the future for vaccination and research against this viral disease. 

Never has a vaccine been successfully developed so quickly against an emerging disease [3,4]. Billions of people have been vaccinated against COVID-19 using mRNA vaccines and more conventional approaches [5]. However, there is still room for improvement regarding the vaccines and the public acceptance of the injections in the human populations in its all diversity. First, because of the fast-evolving nature of coronavirus genomes and the continuous emergence of new variants, close monitoring of vaccine effectiveness and the development of upgraded versions of the vaccines able to control the new mutations are needed [6,7,8]. Recently, Moderna announced promising results for mRNA-1273.214 eliciting immunity against the Omicron variant [9], and there is no doubt other companies will follow up soon with updated versions of their vaccines. Additionally, mucosal vaccination and the development of vaccines that can be delivered through nasal and oral routes are progressing. Currently, there are two ongoing trials assessing parenteral–mucosal strategies for SARS-CoV-2 vaccination using spike and nucleocapsid proteins: NCT04732468 and IG/VPIN/CVD19/2001 [10]. Furthermore, in France, the development of a candidate vaccine that could be administered nasally [11] is progressing well and in July 2023 a phase I/IIa study will be started to evaluate its safety, tolerability, and immunogenicity. The ability of mucosal vaccines to elicit an early antiviral response preventing systemic circulation of viral particles could be a breakthrough in the fight against the virus and could have a significantly greater impact on SARS-CoV-2 infections than the current vaccines. This new generation of vaccines could also be more easily accepted by the general population than the parenteral vaccines for which the fear of injection remains significant. The determinants of vaccine hesitancy have been analyzed in many studies in different contexts and in various situations in our journal in recent months [12,13,14,15,16,17,18]. A deep understanding of these hesitancy determinants and how we can act on them would definitely contribute to increasing vaccination coverage in the general population. This increase is crucial, especially for fragile populations and for immunocompromised individuals.

Another challenge in the field of COVID research is to decipher all the aspects of the immune response developed after vaccination, especially the cellular immune response [19] which still remains more challenging to monitor than its humoral counterpart. However, recent progress has been made, and humoral as well as cellular immune responses to the four main anti-COVID-19 vaccines have gained a more complete understanding [20,21,22]. More specifically, in a recent study [20] it was shown that the mRNA vaccines were the most immunogenic for all the antigen-specific immune metrics analyzed. The mRNA vaccines were associated with significant declines in the neutralizing antibody titers in the initial 6 months following vaccination, while memory CD4+ and CD8+ T cells exhibited small reductions and memory B cells showed small increases [20]. In addition to this progress in our understanding of the immune responses developed after vaccinations as well as following natural infections, many questions remain unanswered. There is still much to discover about long COVID [23] and the complex and various interactions between SARS-CoV-2 and all its target cells requires more research. There is no doubt the coming years will bring exciting new findings regarding SARS-CoV-2 pathophysiology.

SARS-CoV-2 is more and more frequently directly or indirectly detected in different animal species, and many reports have shown its capacity to productively infect various animal species including the white-tailed deer (*Odocoileus virginianus*) [24,25] and the American mink (*Neovison vison*) [26]. The potential development of animal reservoirs and the risk of re-emergences, and even of emergences of significantly mutated strains, could suggest at some point a need to vaccinate some animal species against SARS-CoV-2 (and/or related viruses). Most of the emerging pathogens come from animal species [27,28,29], and a close monitoring of these species, with possibly one health prophylactic or therapeutic intervention [29], is required, ideally before the emergence itself. Thus, vaccination against SARS-CoV-2 and more generally against coronaviruses in animals has a promising future. In humans, a second generation of vaccines against COVID-19 is coming, and in June 2022 the pediatric version of the anti-COVID-19 vaccine has been approved for children in the USA [30]. Age-specific [30] and medical condition-specific vaccination procedures and formulations [31,32,33,34,35,36,37,38,39] definitely constitute a new paradigm in the “vaccine world”. Major developments are expected in the near future. Indeed, mRNA vaccines are fantastic tools to manage the COVID crisis [20]. However, these tools can be further improved to decrease supply logistics issues and to generate longer and more universal immune responses. Self-amplifying mRNA and new delivery platforms, for instance, are in the pipeline, along with mucosal vaccine candidates, to provide populations with new generations of vaccines against SARS-CoV-2 infections and COVID-19 disease.

To conclude, the COVID-19 crisis has been impressive and inspiring for immuno-virology and vaccine development with the official beginning of an mRNA vaccine era for mass vaccination. After a one-year battle, the virus spread is still not fully controlled, and the virus has not yet been defeated. Many challenges still need to be addressed by scientists and health specialists. Moreover, disparities and inequalities between countries are still significant barriers to disease control, underscoring the urgent need for reliable, efficient, and affordable COVID-19 vaccines in many countries.
